# Anomalous Circumrenal Inferior Vena Cava Associated With Horseshoe Kidney

**DOI:** 10.7759/cureus.18797

**Published:** 2021-10-15

**Authors:** Aaiz Hussain, Elizabeth Han, Robert Colvin, Sayf Al-Katib

**Affiliations:** 1 Nova Southeastern University, Dr. Kiran C. Patel College of Allopathic Medicine, Davie, USA; 2 Department of Radiology, Hospital of the University of Pennsylvania, Philadelphia, USA; 3 Department of Diagnostic Radiology and Molecular Imaging, Beaumont Health, Royal Oak, USA

**Keywords:** inferior vena cava anomaly, horseshoe kidney stones, hydroureteronephrosis, ct (computed tomography) imaging, horseshoe kidney, inferior vena cava

## Abstract

A 69-year-old male presented with periumbilical pain radiating across his abdomen, with associated nausea and emesis. CT imaging of his abdomen and pelvis revealed calculi in the right and left ureterovesical junctions with hydroureteronephrosis bilaterally. Furthermore, the imaging revealed that the patient had a horseshoe kidney with an associated anomalous inferior vena cava (IVC) that split superiorly to the horseshoe kidney at the L1 level and rejoined inferior to the horseshoe kidney at the L5 level. The IVC took on a “circumrenal” course, as it traversed the right kidney with an anterior and posterior portion. Furthermore, the patient’s right ureter was compressed between the anterior portion of the IVC and the right kidney. We hypothesize that the development of the horseshoe kidney around the 7 to 8th week of gestation created a path of resistance for the forming of IVC around the same time. While surgical correction is not warranted, recognition of this circumrenal IVC variant could have major implications for planning of procedures, such as IVC filter placement.

## Introduction

Abdominal pain is one of the most common complaints of patients presenting to the emergency department. We present a case of a patient who presented with periumbilical pain, with associated nausea and emesis. CT imaging of his abdomen and pelvis was done and saw nephrolithiasis associated with the patient’s horseshoe kidney. About 20% to 80% of patients with horseshoe kidney can present with kidney stones [1]. However, imaging also found a rare and never-before reported anatomical abnormality pertaining to his inferior vena cava (IVC). What makes this case further unique and interesting is the anomalous IVC’s relationship with the patient’s horseshoe kidney.

## Case presentation

**Figure 1:**
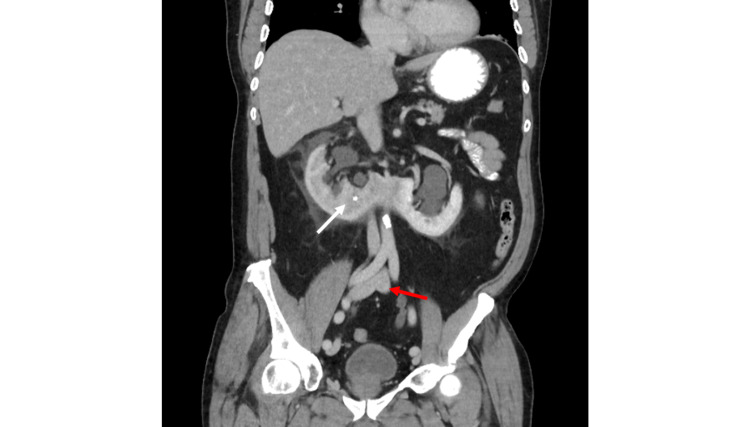
Coronal contrast-enhanced CT with intravenous and oral contrast demonstrates horseshoe kidney with nephrolithiasis bilaterally (white arrow). Of note, the iliac vein confluence is at the pelvic inlet, which is at the level of the S1 vertebral body (red arrow).

A 69-year-old male presented to the emergency department with periumbilical pain radiating across his abdomen, with associated nausea and emesis. CT abdomen and pelvis with intravenous and oral contrast revealed an obstructing 5 mm ureteral calculus in the right ureterovesical junction causing severe hydroureteronephrosis of the right portion of the patient’s horseshoe kidney, a delayed right nephrogram, and severe right perinephric stranding. Additionally, there was a 1.3 cm ureteral calculus in the left ureterovesical junction, causing moderate hydroureteronephrosis of the left portion of the horseshoe kidney and mild perinephric stranding. There were also bilateral non-obstructing renal calculi in the horseshoe kidney (Figure 1).

**Figure 2:**
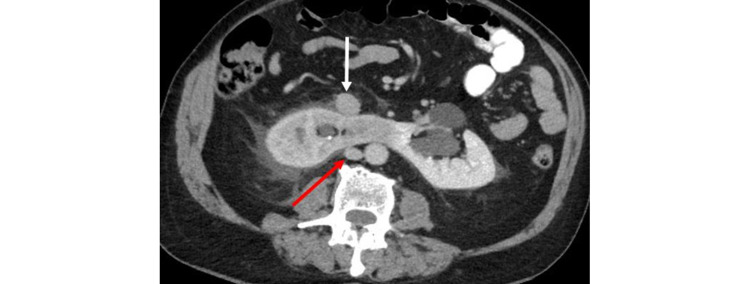
Axial contrast-enhanced CT with intravenous and oral contrast demonstrates a horseshoe kidney with an anterior pre-isthmic IVC (white arrow) and a posterior IVC (red arrow). Additionally, there is bilateral hydroureteronephrosis from bilateral obstructing ureterovesical calculi with a delayed right nephrogram and bilateral perinephric stranding, severe on the right and mild on the left. IVC: inferior vena cava

**Figure 3:**
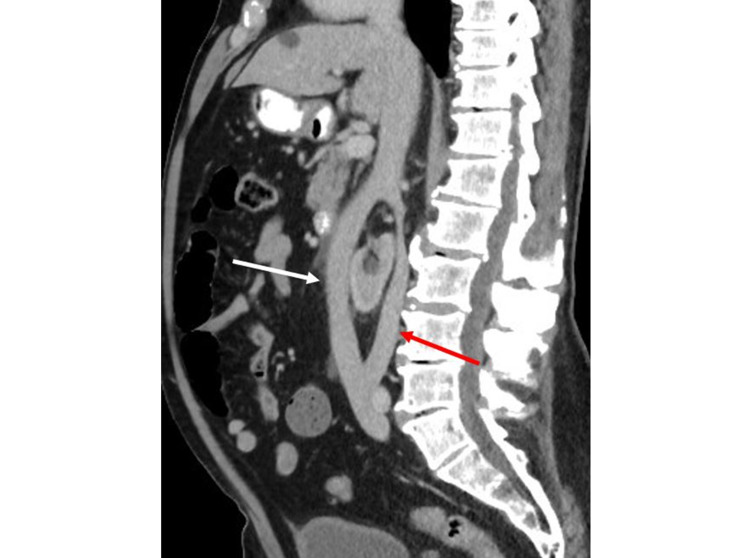
Sagittal contrast-enhanced CT with intravenous and oral contrast demonstrates the split of the IVC at the level of the L1 vertebral body with a pre-isthmic IVC (white arrow) and a posterior IVC (red arrow) which closely approximates the horseshoe kidney before rejoining at the level of the L5 vertebral body. IVC: inferior vena cava

**Figure 4:**
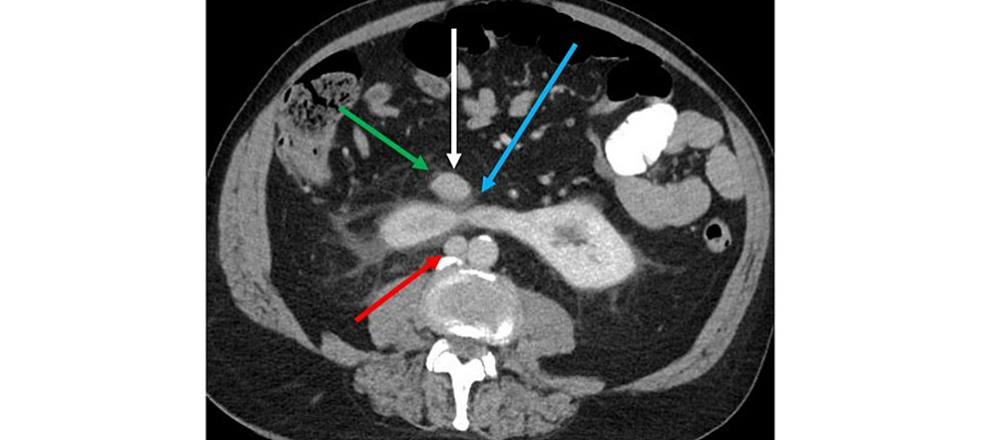
Axial contrast-enhanced CT with intravenous and oral contrast demonstrates a horseshoe kidney with a pre-isthmic IVC (white arrow) and a posterior IVC (red arrow). Right gonadal vein (green arrow) drains into the pre-isthmic IVC. Narrowed right retrocaval ureter (blue arrow) between the pre-isthmic IVC and the horseshoe kidney. IVC: inferior vena cava

While the ureteral and renal calculi accounted for the patient’s presenting symptoms of abdominal pain, the CT scan also revealed the horseshoe kidney having an associated anomalous IVC. The IVC had a “circumrenal” course surrounding the horseshoe kidney. The IVC split above the horseshoe kidney at the level of the L1 vertebral body into a pre-isthmic IVC (anterior to the horseshoe kidney) and a posterior IVC (Figure 2). Below the kidney, the IVC rejoined at the level of the L5 vertebral body. On sagittal CT imaging, the IVC closely approximates the anterior and posterior aspects of the horseshoe kidney (Figure 3). The right gonadal vein was noted to drain into the pre-isthmic IVC. Additionally, the right ureter took a retrocaval course posterior to the pre-isthmic IVC. There was narrowing of the right retrocaval ureter between the horseshoe kidney and the pre-isthmic IVC (Figure 4).

## Discussion

Anomalies of the IVC were first described in 1793, in a 10-month-old child who had dextrocardia and polysplenia. He presented with a congenital mesocaval shunt with continuation of the IVC with the azygos vein [2]. With the widespread use of cross-sectional imaging, multiple congenital anomalies of the IVC in asymptomatic patients have been incidentally noted since then [3]. The prevalence of IVC anomalies is reported at 0.5% in the world [2]. It has been suggested that there may be up to 14 variations in the anatomy of the IVC [3].

The formation of the IVC can be traced to 4 to 8 weeks of gestation. First, the posterior cardinal veins develop and form the distal IVC, which consists of the iliac bifurcation. Afterwards, two subcardinal veins are formed. The right subcardinal vein eventually becomes the suprarenal IVC, whereas the left subcardinal vein regresses. Finally, two supracardinal veins develop. The right supracardinal vein forms the infrarenal IVC, and the left supracardinal vein regresses [4]. Common congenital anomalies of the IVC include: 1) left IVC: the IVC is on the left side and then joins the right renal vein to form a right prerenal IVC, 2) azygos continuation of the IVC: the renal portion of IVC has blood return from the kidneys, and the IVC courses posteriorly to the diaphragmatic crura, and then enters the thorax as the azygos vein, with the azygos vein joining the IVC in the right paratracheal space, 3) circumaortic left renal vein: there are two left renal veins, one left renal vein courses posteriorly to the abdominal aorta and the other left renal vein courses anteriorly to the abdominal aorta before joining the right-sided IVC, 4) retrocaval ureter: the right ureter courses between the right kidney posteriorly and the IVC anteriorly, and 5) double IVC: duplication of the IVC, where the left IVC drains into the left renal vein, which then joins the right IVC, and results from persistence of both the right and left supracardinal veins [3, 5].

Horseshoe kidney is a congenital anomaly that occurs in 0.25% of the general population. In patients with a horseshoe kidney, congenital anomalies of the IVC increase to 5.7% [6]. The known anatomical IVC variations in the horseshoe kidney are pre-isthmic IVC where the IVC lies anterior to the horseshoe kidney, double IVCs which are posterior to the horseshoe kidney, left IVC which is posterior to the horseshoe kidney, and azygos continuation of the IVC [6, 7]. To the authors’ knowledge, there is no previously reported case in the literature that describes a horseshoe kidney with an IVC that has a course “circumrenal” to the horseshoe kidney having a pre-isthmic IVC and a posterior IVC, both on the right side of the horseshoe kidney. We have coined the term “circumrenal” as the IVC splits and appears to hug the horseshoe kidney, which is analogous to a circumaortic left renal vein that hugs the abdominal aorta.

There are two theories to the embryological development of the horseshoe kidney. The theory of mechanical fusion suggests that a horseshoe kidney occurs due to contact of the metanephric blastema of the kidneys in the 4th week of embryogenesis. When this contact is made, the blastema of the kidneys fuse due to the lack of a renal capsule. This results in formation of the fibrous isthmus. When the kidney ascends in the 7th to 8th week, the isthmus gets trapped under the inferior mesenteric artery. Another theory suggests that the horseshoe kidney occurs from faulty migration of posterior nephrogenic cells which causes formation of the isthmus [8]. The kidney ascends and places itself under the inferior mesenteric artery around the 7th to 8th week and this is also when the IVC is forming. Thus, the development of our patient’s “circumrenal” IVC may be tied to the ascension of the horseshoe kidney. We hypothesize that the horseshoe kidney’s placement within the pelvis created a path of resistance for the formation of the IVC. The resulting “circumrenal” IVC may be due to fusion of the posterior cardinal vein and the supracardinal vein above and below the horseshoe kidney. Thus, it could be concluded that this circumrenal anomaly only occurs in patients with horseshoe kidney.

While most patients with IVC anomalies have been found to be asymptomatic, in some young patients, deep vein thrombosis, varicocele, and varicose veins have been observed. The main clinical consideration for patients with IVC anomalies is pre-surgical and interventional procedure planning, for example, for IVC filter placement [9]. In this case, our patient presented with obstructing bilateral ureteral calculi causing severe abdominal pain where the narrowed right retrocaval ureter may have been a contributing factor. Similar symptoms of hydronephrosis and calculi can be observed in retrocaval ureter anomalies where the right ureter courses posterior to the IVC. Retrocaval ureters develop embryologically in the 4th to 8th week of gestational period, similar to other IVC anomalies, and clinically present in the 3rd to 4th decade of life. Only 200 cases of retrocaval ureters have been reported, but present with trapping and compression of the right ureter with associated symptoms. This compression has been treated with surgery with resection of the part of the ureter coursing posterior to the IVC and transposition of the ureter anterior to the IVC [10]. Our patient, presenting with his own unique version of a retrocaval ureter, was not treated surgically, but instead underwent a right ureteral stent placement by the urology team. The urology team was not able to place a left ureteral stent. As a result, the patient had placement of a left percutaneous nephrostomy tube and a nephroureteral stent by the Interventional Radiology team. He was treated with antibiotics and discharged. One month after discharge, the patient underwent laser lithotripsy and stone basketing. The non-invasive nature of stent placement is indicated for treatment of bilateral obstruction of the kidneys from internal and external causes [11]. The internal cause, in this case, is the calculi bilaterally and the external cause is the compression of the right ureter by the IVC. The long-term success of stents for our patient’s symptoms remains to be seen.

## Conclusions

This case describes a patient with a never-before reported anatomical variation of the IVC. This “circumrenal” course of the IVC encompassed the patient’s horseshoe kidney. We hypothesize that the development of the horseshoe kidney around the 7th and 8th weeks of gestation, most likely created a path of resistance for the developing IVC around the same time. The clinical significance of this anatomical variation is twofold. The first is the compression of the patient’s right ureter between the anterior portion of the IVC and right portion of the horseshoe kidney which may have contributed to the development of renal calculi and hydroureteronephrosis. The second is the significant impact on potential procedural planning and the adjustments necessary. Our patient’s ureter compression was treated with ureteral stents.
